# Self-Contained Gene-Set Analysis of Expression Data: An Evaluation of Existing and Novel Methods

**DOI:** 10.1371/journal.pone.0012693

**Published:** 2010-09-17

**Authors:** Brooke L. Fridley, Gregory D. Jenkins, Joanna M. Biernacka

**Affiliations:** Department of Health Sciences Research, Mayo Clinic, Rochester, Minnesota, United States of America; University of Minnesota, United States of America

## Abstract

Gene set methods aim to assess the overall evidence of association of a set of genes with a phenotype, such as disease or a quantitative trait. Multiple approaches for gene set analysis of expression data have been proposed. They can be divided into two types: competitive and self-contained. Benefits of self-contained methods include that they can be used for genome-wide, candidate gene, or pathway studies, and have been reported to be more powerful than competitive methods. We therefore investigated ten self-contained methods that can be used for continuous, discrete and time-to-event phenotypes. To assess the power and type I error rate for the various previously proposed and novel approaches, an extensive simulation study was completed in which the scenarios varied according to: number of genes in a gene set, number of genes associated with the phenotype, effect sizes, correlation between expression of genes within a gene set, and the sample size. In addition to the simulated data, the various methods were applied to a pharmacogenomic study of the drug gemcitabine. Simulation results demonstrated that overall Fisher's method and the global model with random effects have the highest power for a wide range of scenarios, while the analysis based on the first principal component and Kolmogorov-Smirnov test tended to have lowest power. The methods investigated here are likely to play an important role in identifying pathways that contribute to complex traits.

## Introduction

With the advent of high-throughput technologies, such as microarrays, complete genome-wide studies of genomic predictors of diseases have become common. Many diseases or phenotypes are expected to involve complex relationships of gene products within the same molecular pathway or functional gene set. Therefore, pathways or gene sets, as opposed to single genes, may better reflect the true underlying biology and may be more appropriate units for analysis. Pathway or gene set methods for analysis of expression data incorporate prior biological knowledge into the statistical analysis by evaluating the overall evidence of association of a phenotype with expression of all genes in a given pathway or gene set. Application of such methods may enable the detection of more subtle effects of multiple genes in the same pathway that may be missed by assessing each gene individually. Moreover, the incorporation of biological knowledge in the statistical analysis may aid researchers in the interpretation of results.

Within the last few years, multiple approaches for gene set analysis have been proposed for both expression and SNP data. The various methods can be divided into two types: competitive and self-contained [1]. Competitive methods compare the results for genes within the gene set with results for genes outside the gene set (complement) to determine whether genes in a particular gene set are associated more with a phenotype as compared to genes outside the gene set. Two widely used competitive gene set methods for analysis of gene expression studies are gene set enrichment analysis (GSEA) [2], which uses a Kolmogorov–Smirnov test, and DAVID [3], which uses a Fisher's exact test. Self-contained methods, in contrast to competitive methods, only consider results within a pathway or gene set of interest. Because competitive methods require a comparison between results within a gene set to those outside the gene set, these tests cannot be applied in a study that only measured expression in a particular candidate pathway or gene set. In contrast, self-contained methods can be used for genome-wide studies as well as candidate gene or pathway studies. For more discussion on existing methods for gene set analysis, we refer the reader to Goeman and Buhlmann [1] and Allison et al [4].

Let *S* represent a gene set of interest and *S^C^* represent the complement of *S*. The null hypothesis of self-contained gene set methods is *H_o_^SC^*: *Gene expression levels of all genes in S are NOT associated with the phenotype*, while the null hypothesis for competitive methods is *H_o_^C^*: *Genes in S are associated with the phenotype as much as genes in S^C^*. The self-contained approaches are more powerful for testing the *H_0_^SC^* hypothesis and allow for subject-level sampling or permutation methods for estimating the empirical null distribution of the test statistic, while the competitive methods do not [1].

Liu et al [5] compared three self-contained methods for binary phenotypes: the Global Test of Goeman et al. [6], that involves a global model with random effects, the ANCOVA Global Test of Mansmann and Meister (2005) [7] and SAM-GS (2007) [8], and found that SAM-GS slightly out-performed the Global Test and the ANCOVA Global Test. Dinu et al [9] also compared five self-contained gene set methods and the competitive GSEA method [2] using three microarray studies and concluded that the self-contained methods of SAM-GS, Global test and ANCOVA Global outperformed GSEA. However, general conclusions regarding the relative performance of the investigated methods could not be made, as no simulation studies were completed. Although SAM-GS is only applicable to data with binary endpoints, an extension of SAM-GS recently introduced by Adewale et al [10] is applicable to diverse phenotypes, including binary, multiclass, continuous, and survival endpoints. Tsai and Chen [11] proposed using a MANOVA test for gene-set analysis and compared it to several methods including principal component analysis, SAM-GS, GSEA, MaxMean, analysis of covariance, and a global test. Based on simulation results and real data analysis they found the MANOVA approach appeared to perform best, but concluded that most methods, except GSEA and MaxMean were generally comparable in terms of power. A limitation of the MANOVA method of Tsai and Chen [11] (2009) is that it is only applicable to categorical outcomes data.

In this paper, we present an extensive study of ten methods for conducting a self-contained gene set analysis to test the hypothesis *H_0_^SC^*. Using the most extensive simulation study to date (over 2000 simulation scenarios and ten self-contained gene set analysis approaches), we investigated previously proposed methods and a newly proposed method, which combines the ideas of Fisher's Method [12] and Tail Strength [13]. We limited our study to self-contained gene set methods that can be used for continuous, binary or time to event endpoints/phenotypes, and allow for the inclusion of covariate information. The following gene set methods were assessed: Kolmogorov–Smirnov test (KS), Fisher's method (FM), Stouffer's method (SM), tail strength (TS), a novel modified tail strength statistic (MTS), global model with fixed effects (GMFE), global model with random effects (GMRE), and principal component analysis (PCA). These methods can be divided into those based on summaries of results for individual expression probes (e.g., p-values) (FM, SM, KS, TS, MTS) and those based on joint modeling of all data for a given pathway (PCA, GMRE, GMFE). The simulation scenarios varied according to: number of genes in a gene set, number of genes associated with the phenotype, effect sizes, correlation between expression of genes within a gene set, and the sample size.

In the following sections we briefly describe the various self-contained gene set methods for analysis of gene expression data and provide details of the data simulation. We then present results from the extensive simulation study that assessed the statistical performance of the various gene set methods. For illustration of the methods, we also present results from a pharmacogenomic study of the drug gemcitabine.

## Materials and Methods

### Gene set analysis methods based on association p-values

In following sections we describe several gene set approaches for testing the null hypothesis *H_0_^SC^* (expression levels of all genes in a gene set are not associated with the phenotype) that use p-values from separate tests of association of the phenotype with the expression of each gene in the gene set. All of these approaches rely on first testing for association between the phenotype and the expression measured by each of *m* probes individually. Let *p*_i_ represent the p-value from the test of the null hypothesis *H_oi_*, *i* = 1, 2, …, *m* (*H_oi_*: expression measured by i^th^ probe is not associated with the phenotype). The tests used to calculate *p_i_*'s will depend on the type of phenotype. For example, for a continuous phenotype, such as glucose level, the p-values for association with gene expression could be based on a linear regression model. Any appropriate parametric or non-parametric method can be used to calculate the *p_i_*'s; however, power for the GSA will dependent on the use of a powerful method for analysis of data for individual expression probes. The p-values for these *m* tests are then used to calculate a single test statistic and p-value for testing *H_0_^SC^*.

Throughout this paper **Y** denotes a vector of size *n*×*1* that contains continuous phenotypic values for the n subjects, and **X** represents a matrix of size *n*×*m* that contains the gene expression values for the *m* genes within the gene set *S* measured on the *n* subjects.

#### Kolmogorov-Smirnov test

The Kolmogorov-Smirnov (KS) test is a goodness of fit test that determines if a given empirical distribution, F(x), differs significantly from a hypothesized distribution, F_o_(x). KS is one of the most useful and general nonparametric methods for testing goodness of fit, as it is sensitive to differences of the empirical cumulative distribution function from the hypothesized function, in both location and shape. In the context of testing *H_0_^SC^*, the KS test for goodness of fit can be applied to determine if the distribution of the observed p-values differs from the distribution under the “null” hypothesis. In particular for gene set analysis, we are interested in testing if the observed p-values are smaller than expected under the null distribution using the one-sided test statistic 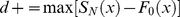, with 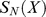 representing the cumulative step-function of a sample. Significance for *d+* can be assessed using the Kolmogorov distribution or Monte Carlo methods. We applied the KS test using a Uniform(0,1) reference (null) distribution; however, to avoid reliance on the null U(0,1) distribution of probe-specific p-values, we used Monte Carlo simulations to determine significance level of the KS-test statistic *d+*.

#### Fisher's method

Fisher's method (FM) [12] and variations of Fisher's method [14], [15], [16], [17] have been used extensively for combining results from multiple statistical tests. See Zaykin et al [16] for a brief review of modifications of Fisher's method for combining p-values. The original Fisher's method for combining independent p-values is based on the fact that since under the null hypothesis *p*_i_∼Uniform(0,1), −2log(*p*_i_) follows a Chi-squared distribution with 2 degrees of freedom (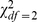). Therefore, the test statistic 
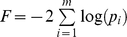
 
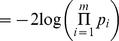
 follows a Chi-squared distribution with degrees of freedom 2*m (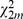). It has been shown that this method of combining p-values from independent tests is asymptotically optimal (among essentially all methods) [18]. As opposed to relying on the asymptotic distributions that assume independent p-values, we used a Monte Carlo approach to determine the empirical p-values of the FM tests.

#### Stouffer's method

Like Fisher's method, Stouffer's Method (SM) [19] is also based on a sum of transformed p-values. However, rather than using the log transformation of Fisher's method, Stouffer (1949) proposed using a normal transformation, and defined the test statistic as 
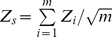
, with 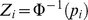, where 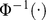 is the inverse standard normal cumulative distribution function [19]. In the setting in which the *m* tests are dependent (which will often be the case in gene set analyses), permutation procedures can be utilized to estimate the distribution of the test statistic. Again, to avoid reliance on the asymptotic distribution of the gene-set association statistic, which assumes independent probe-specific p-values, we used a Monte Carlo approach to determine the empirical p-values of the SM test.

#### Tail strength

The measure of tail strength (TS) proposed by Taylor and Tibshirani [13] can also be used to perform self-contained gene set analysis. Let p_(1)_≤p_(2)_ …≤p_(m)_ denote the ordered p-values (*p_i_*,) for the *m* expression probes in a gene set. Next, define the tail strength as 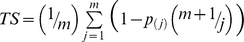. Under the null hypothesis, each p*_m_*∼Uniform(0,1), yielding 
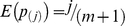
 and therefore E(TS) = 0. As discussed by Taylor and Tibshirani (2006) [13], the tail strength measures the deviation of the ordered p-values from the expectation under the null hypothesis of no association. If overall the ordered p-values are smaller than their expectation, TS >0 providing evidence against the null hypothesis.

Since the statistic TS involves a summation, TS is approximately normal under the null hypothesis when *m* is large and the p-values are independent. In practice, *m* may not be large (<20). In addition, the assumption of independence between gene expressions within a given pathway may not be valid. Thus, we use permutations to determine the empirical distribution of TS under the null hypothesis as a means for determining the p-value for each pathway.

#### Modified tail strength

We propose a modification of the tail strength method, by applying a log-transformation to p-values, as is done in Fisher's method. Let *X_i_* = −log(p_(i)_), where p_(i)_ are the ordered p-values for the *m* expression probes, and define 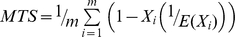. Under the null hypothesis of no association, the random variable 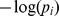 follows an exponential distribution with rate parameter 1. Therefore, the probability distribution of the order statistic *X_i_*, is 
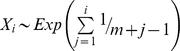
, with expectation 
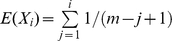
. Under the null hypothesis, the expectation of the test statistic MTS is 0, E(MTS) = 0. In a similar fashion as tail strength [13], the MTS measures the deviation of each ordered −log(p-value) from the expectation under the null hypothesis of no association. However, by using the −log(p-value), as opposed to the p-value as done in the original Tail Strength method, we hope to gain power for testing gene set effects. Since the assumption of independence between genes within a given pathway may not be valid, we again use permutations to determine the empirical p-value of MTS for each pathway.

### Gene set analysis method based on data modeling

Rather than relying on individual gene (probe set) p-values, *H_0_^SC^* can also be tested by jointly modeling the effects of expression of genes within a pathway. The methods described in following three sections.

#### Global model using fixed effects

Possibly one of the simplest methods for completing a gene-set analysis is to fit a regression model (linear, generalized linear, or Cox-proportional hazards model) in which the expression levels of all *m* genes in the gene set are included in the model as fixed effects (GMFE). Assessment of association of the phenotype with the gene set is then based on testing the null hypothesis 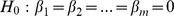 versus the alternative that at least one of the coefficients differs from zero [20], [21], [22]. A limitation of this approach is that the model is only estimable when *m*<*n*.

#### Global model using random effects

The global model approach of Goeman et al [6] is based on a linear random effects model in which the continuous phenotype is modeled as a function of the expression values for the genes within the gene-set of interest (GMRE). That is, **Y** is modeled as 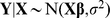 with the gene expression effects, 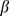, having a common distribution with mean 0 and variance 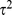. Under the null hypothesis of no gene expression effects on the phenotype, the variance of the expression effects is zero (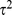 = 0), which can be tested with a score test [6]. This score test is locally optimal for 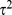 = 0 [23]. The method can be modified to include covariates and/or other phenotypic types (e.g., binary, survival, multi-class).

#### Principal components analysis

Principal Component Analysis (PCA) is a broadly-applicable dimension-reduction technique [24]. The basic goal of PCA is to reduce the dimension of the data, which is accomplished by choosing *p* components instead of the total set of *m* variables (gene expression), where *p*≪*m*. The components are linear combinations of the original predictor variables. Gene set analysis using PCA was completed in a manner similar to that described previously [17], [24], [25]. In gene expression data analysis, the principal components are created using linear combinations of gene expression values. Previous applications of PCA to gene set analysis used either only the first principal component [25], [26] or the top 3 to 5 principal components [17], [27]. We used the top *k* principal components needed to explain 80% (PCA80) of the variation in the gene expression values within the gene set, along with the methods based on the top one (PCA1) or top five principal components (PCA1.5). The gene set association analysis is then based on a likelihood ratio test for the effect of all the PCs included in the model.

### Permutation-based assessment of significance of gene set association

Non-independence of gene-specific p-values due to correlation of expression of genes in a gene set, and other factors such as small sample size, can lead to departures of p-values from the expected Uniform(0,1) distribution, even in the absence of expression-phenotype association. Therefore, rather than relying on asymptotic distributions of the gene-set association statistics for the KS, FM, SM, TS, MTS and GMRE methods, we use permutations to obtain gene-set association p-values for these methods. First, the phenotypic or response variable is randomly permuted keeping the gene expression data fixed (and thus keeping the correlation structure in the expression data). An association test for each gene within the gene set is then computed based on the data set with the permuted phenotype, and the gene set analysis statistic and corresponding asymptotic p-value is calculated. This process is repeated many times (e.g. 1,000 times), producing an empirical distribution of the gene-set test statistic (and corresponding p-values). The proportion of permutations in which the gene set analysis p-value is smaller than the gene set p-value in the original data provides the empirical estimate of the p-value for the gene set test for association. Thus, although we use previously proposed statistics, such as the F-statistics proposed by Fisher [12], as summary statistics for a pathway, by using permutation to assess significance of these statistics we remove the requirement for specific null distributions (such as the assumption of the chi-square distribution for the F-statistic). Therefore, none of the approaches rely on the assumption that the probe-specific p-values, p_i_, are uniformly distributed under the null hypothesis. Furthermore, there is no assumption of independence of probe-specific p-values.

### Data

#### Simulation Study

To assess the power and the type I error rate for the various self-contained gene set approaches, an extensive simulation study was completed. The simulated scenarios varied according to: number of genes in a gene set, number of associated genes, effect sizes for the associations, correlation between expression of genes within a gene set, and the sample size. For each scenario, 1000 simulated datasets were generated to estimate power or type I error rate.

Let *n* represent the number of subjects, and *m* represent the number of genes (or expression probe sets) in a gene set. The expression data for each subject was simulated from a multivariate normal distribution, 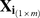
∼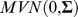 *i* = 1,…,*n*. The covariance matrix 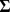 was set to be either one in which there is no correlation of expression values in a pathway so that 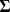 is diagonal, or a structure in which all genes in the pathway are correlated the same amount, i.e. exchangeable (with correlation *ρ* = 0.1 or 0.3 and variance set to 1). Next, the quantitative phenotype (*Y*_i_) for each subject was generated conditional on their gene expression values, 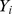
∼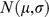 with 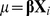. The specification of the effect sizes 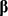 was varied according to the simulation scenario, with 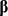 = 0, 1, 2, or 3 indicating: no, small, medium, and large effect size.

In total, 114 “null” scenarios, with no association of expression at any gene in the pathway, were simulated to investigate type 1 error for a range of sample sizes (n = 20, 100 or 500), gene set sizes (m = 10, 50, 100, or 500), error standard deviation (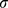 = 1, 3, or 6) and levels of correlation between expression levels at different genes (*ρ* = 0, 0.1 or 0.3). For the null scenarios all 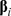 = 0. Details of the simulation scenarios investigated are presented in **Table S1**. In addition, 2268 scenarios were generated with gene expression for at least one probe associated with the quantitative trait, to investigate power of the gene set analysis methods. Simulation settings for all “non-null” scenarios are presented in **Table S2**. As for the type 1 error simulations, sample size, gene set size, error variance and correlation between expression at different probes were varied for the power analyses. In addition, the number of genes with expression associated with the quantitative trait, and the effect sizes for these associations, were varied, by specifying a non-zero vector of effect sizes, 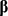.

In addition, we completed simulations with the simulated data having similar correlation patterns to real data from a pharmacogenomic study of gemcitabine (described in subsequent section). Gene expression data were simulated using the correlation structure of the observed data, consisting of 31 probes within the gemcitabine pathway. For these simulations, gene set size was therefore fixed at 31. The phenotype was simulated under the models outlined above, restricted to scenarios with the number of effects being less than 31 probes. Sample sizes of 100 and 500 were considered. **Table S2** includes the 108 non-null simulation scenarios based on the gemcitabine pathway correlation structure. Similarly, **Table S1** includes the 6 null simulation scenarios with this correlation structure.

All simulated data sets were analyzed with the various self-contained gene set methods. A test for the association of the continuous phenotype with expression was calculated for each probe using an F-statistic for the correlation between the gene expression and phenotype, i.e. 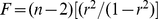. P-values were computed based on the F distribution with degrees of freedom 1 (numerator) and *n*−2 (denominator). Empirical p-values for the KS, FM, SM, TS, MTS and GMRE methods were all based on 1,000 permutations. Results for data generated under the null hypothesis of no gene set association were used to assess type 1 error rate, while results for data generated under the alternative hypothesis were used to assess power. Power and type 1 error rates were estimated for all methods based on a 0.05 significance level. In addition to comparing mean power across scenarios between methods, we investigated trends in power as a function of characteristics of the simulation scenarios (e.g. sample size, effect size). To summarize the extent of genetic contribution of a gene set to the phenotype, we calculated R^2^, the proportion of variation in the quantitative phenotype due to gene expression variation. R^2^ was calculated as 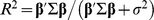. All analyses were completed in R with code available from the authors upon request.

#### Pharmacogenomic study of gemcitabine

Pancreatic cancer is a rapidly fatal disease with a 5-year survival rate of less than 5%. However, drug response to the standard chemotherapy for pancreatic cancer, gemcitabine, varies widely among individuals. Host variation in transport, metabolism and targets for drugs used to treat pancreatic cancer may influence clinical response to therapy. Variation in genes that play a role at each step within the gemcitabine metabolic pathway could potentially influence the quantity of drug transported into the cell, metabolic inactivation of the drug, the rate of active drug formation and the quantity of active drug reaching its target(s).

To develop hypotheses to be tested in translational studies involving pancreatic cancer patients, cytotoxicity studies using a cell-line model system were performed as previously described [28]. Gemcitabine cytotoxicity data were collected at eight drug dosages (e.g., 1000, 100, 10, 1, 0.1, 0.01, 0.001, and 0.0001 uM) for 194 cell lines, and the phenotype IC_50_ (effective dose that kills 50% of the cells) was estimated using a four parameter logistic model. In addition to IC_50_ phenotypic data, whole genome expression data for these cell lines was obtained with the Affymetrix U133 plus 2.0 expression array chip, which contains over 54,000 probe sets designed based on build 34 of the Human Genome Project. The mRNA expression array data were normalized on the log_2_ scale using GCRMA methodologies [29], [30], [31], and association of IC_50_ with expression of each probe was investigated (see Li et al [28] for details of experimental design, analyses and results).

In the previous analyses reported by Li et al [28], each expression probe set was analyzed separately; however no gene set analyses were performed. For illustration of the various self-contained gene set methods, we applied the methods to investigate the impact of gene expression in three gene sets on IC_50_. Based on current knowledge of the function of genes in the gemcitabine pathway, these genes are believed to play a role in response to the drug gemcitabine (http://www.pharmgkb.org/do/serve?objId=PA2036&objCls=Pathway). Thus, variation in expression of these genes may impact the IC_50_ phenotype. We therefore chose this pathway to illustrate the application of the methods, expecting that we may obtain evidence of association of this candidate gene set with the IC_50_ phenotype. The gemcitabine pathway contains 31 probe sets from the Affymetrix U133 plus 2.0 expression array. We also applied the various GSA methods to a randomly selected set of 20 genes (“null” gene set) and another important drug metabolizing pathway, the “glutathione” pathway, as this pathway is the major metabolic pathway responsible for acetaminophen-NAPQI detoxification [32], [33] and response to platinum-containing drugs used in the treatment of ovarian and lung cancers [34]. The glutathione pathway contained 52 probe sets from the Affymetrix U133 plus 2.0 expression array. Gene set analysis of these two pharmacogenomic pathways and the null gene set was performed as described for the simulated data. In performing GSA for the gemcitabine cytotoxicity study, empirical p-values for the KS, FM, SM, TS, MTS and GMRE methods were based on 10,000 permutations.

## Results

### Simulation study

**Figure 1:**
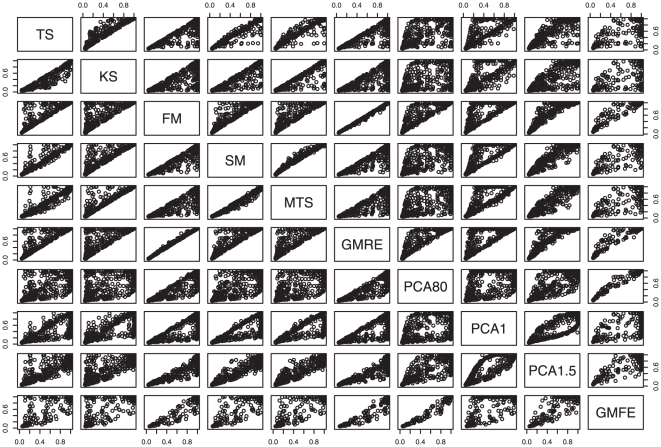
Pairwise scatterplot of power for the various methods for scenarios with standard deviation (σ) of 6.0.

Table 1 shows the mean type 1 error over all “null” scenarios for all methods that were investigated. All methods had correct type 1 error under all simulated scenarios (see **Table S1** for detailed results for each null simulation scenario). **Table S2** shows the power of all methods for all “non-null” simulated scenarios. The mean power across all scenarios is shown in the last column of **Table 1**. **Figure 1** displays pairwise comparisons of power, for all methods, across all simulated scenarios. Looking at the rows of scatterplots corresponding to FM and GMRE, the points fall on or above the diagonal, indicating that these methods have higher power than the other approaches over all scenarios investigated. Consistent with this observation, these two methods had the highest mean power across all scenarios. Although GMFE had even higher mean power under situations when it could be applied, this method was not applicable in most scenarios, particularly those with small sample sizes. For the simulation scenarios investigated here, KS and principal component analysis with the top component (PCA1) had the lowest mean power. With a small sample size of n = 20 all of the principal component methods had low power.

**Table 1:** The mean type 1 error and power for all gene set methods averaged across all null (mean type 1 error) non-null (mean power) simulation scenarios for sample sizes of 20, 100, and 500.

		Mean Type 1 Error			Mean Power		
Type of Method	Gene Set Method	N = 20	N = 100	N = 500	N = 20	N = 100	N = 500
Based on combining individual SNP p-values	Kolmogorov-Smirnov (KS)	0.047	0.048	0.053	0.533	0.751	0.831
	Fisher's Method (FM)	0.048	0.050	0.052	0.608	0.894	0.981
	Stouffer's Method (SM)	0.048	0.049	0.051	0.571	0.825	0.937
	Tail Strength (TS)	0.048	0.049	0.052	0.573	0.807	0.876
	Modified Tail Strength (MTS)	0.050	0.048	0.048	0.549	0.798	0.929
Based on modeling the data	Global model using fixed effects (GMFE)*	0.053	0.045	0.051	0.639	0.907	0.985
	Global model using random effects (GMRE)	0.048	0.050	0.053	0.604	0.900	0.984
	PCA using 1st principal component (PCA1)	0.050	0.050	0.049	0.537	0.717	0.821
	PCA using1–5 principal components (PCA1.5)	0.046	0.050	0.050	0.543	0.800	0.925
	PCA using principal components that explain 80% (PCA80)	0.049	0.047	0.052	0.489	0.861	0.975

*GMFE could not be applied in 27 out of 36, 18 out of 39, and 9 of the 39 simulation scenarios used to assess type 1 error at samples sizes of 20, 100, and 500, respectively. GMFE could not be applied in 594 out of 720, 396 out of 774, and 198 of the 774 power scenarios with sample sizes of 20, 100, and 500, respectively.

Overall, the novel MTS method led to improvements in power over the existing TS method for large sample sizes (n = 500), while having lower mean power for small sample sizes (n = 20). The increase in power was more pronounced for large sample sizes and models with fewer associated expression probes and low correlation between expression probes. Among the PCA methods, analysis based on principal components that explain 80% of the variation (PCA80) had substantially higher power than analysis based on either the top component (PCA1) or the top five components (PCA1.5) for large sample sizes, except when there was a high correlation between expression probes, in which case all the PCA methods had similar performance. However, with the small sample size (n = 20) PCA80 had lowest power among the investigated methods.

**Figure 2:**
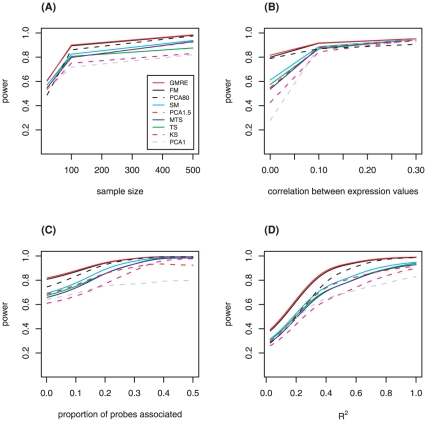
Plots of power for all methods. Power is plotted as a function of (A) sample size, (B) the correlation between expression values within the gene set (*ρ*), (C) the proportion of probes associated with the phenotype, and (D) the calculated R^2^, the proportion of variation in the quantitative phenotype explained by the gene expression values in the pathway. The average power values are based on all simulated non-null scenarios. Plot (B) excludes scenarios with between-probe correlation structure defined by the gemcitabine pathway, and only shows fixed-correlation scenarios (ρ = 0, 0.1, 0.3). Plots (B), (C), and (D) are based on sample size of 100. Similar plots for sample sizes of 20 and 500 are shown in Figure S1. For plots (C) and (D) a kernel smoother was used to fit a curve to the data. Scenarios with all expression probes being associated with the trait were excluded from plot (C), as all the methods had very high power in this situation.

**Figure 3:**
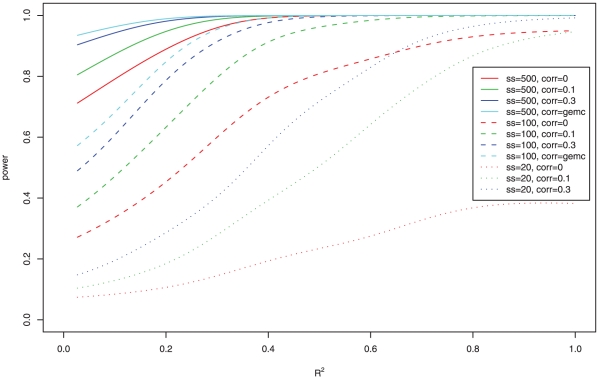
Power of Fisher's Method (FM) as a function of sample size, correlation of expression values between probes (*ρ*), and R^2^ (proportion of variation in the quantitative phenotype explained by the gene expression values in the gene set).

For all methods, power was generally higher at larger sample size (**Figure 2A**), and when the correlation between expression probes was higher (**Figure 2B**). For each simulation model, we also calculated the proportion of probes associated with the phenotype and R^2^, the proportion of variation in the quantitative phenotype explained by the gene expression values in the pathway. As expected, power of all methods increased when more expression probes were associated with the phenotype (**Figure 2C**) and as R^2^ increased (**Figure 2D**). Figures 2B–2D show the relationship of power to the various model parameters (e.g. number of associated probes, R^2^) for sample size of n = 100. Similar plots for sample size of 20 and 500 are shown in **Figure S1**. **Figure 3** shows power of FM as a function of sample size, correlation of expression values between probes, and R^2^, again demonstrating the increase in power with increasing sample size, increasing correlation between probe-specific expression levels, and increasing R^2^. Similar patterns were observed for other methods.

### Gemcitabine pharmacogenomics

The gene set methods investigated by simulations were applied to a pharmacogenomic study of the cancer drug gemcitabine, to examine the impact of the gene set comprised of the gemcitabine pathway, glutathione and null gene set on the drug-related phenotype IC_50_ (effective dose that kills 50% of the cells).

Results of the GSA for the pharmacogenomics data with the gemcitabine pathway, glutathione pathway and null gene set are presented in **Table 2**. For the gemcitabine pathway, evidence of gene-set association varies considerably between the methods, with p-values for the pathway ranging from 0.002 for the PCA1.5 analysis to 0.341 for the PCA1 test. FM, GMRE, GMFE, PCA80 and PCA1.5 provided statistically significant evidence (p<0.05) for association of expression of the gemcitabine pathway gene set with the phenotype IC_50_. Overall, the patterns observed in the analysis of the gemcitabine pathway mimic those patterns observed in the simulation study for large sample sizes (n = 100–500), with PCA1.5, PCA80, FM, and GMRE resulting in the most significant results while PCA1, KS and MTS resulted in the least significant associations. In contrast, the results for the glutathione pathway showed the GMFE approach produced the most significant results (p = 2.27×10−5), while the methods that produced the largest p-values were PCA1 and PCA1.5. The methods based on probe-specific p-value summaries (KM, FM, SM, MTS, TS) all produced similar results (p-values between 0.01 and 0.03). Lastly, all methods, except GMFE and PCA80, produced non-significant results for the gene set containing a random selection of 20 genes.

**Table 2:** Results from analysis of gemcitabine pathway, glutathione pathway and null gene set from the various gene set methods.

Type of Method	Gene Set Method	Glutathione Pathway p-value	Gemcitabine pathway p-value	Null gene set
Based on combining individuals SNP p-values	Kolmogorov-Smirnov (KS)	0.0178	0.250	0.447
	Fisher's Method (FM)	0.0121	0.016	0.126
	Stouffer's Method (SM)	0.0241	0.158	0.272
	Tail Strength (TS)	0.0211	0.160	0.344
	Modified Tail Strength (MTS)	0.0371	0.267	0.278
Based on modeling the data	Global model using fixed effects (GMFE)	2.27×10^−5^	0.032	0.004
	Global model using random effects (GMRE)	0.0137	0.012	0.780
	PCA using 1st principal component (PCA1)	0.3610	0.341	0.668
	PCA using1–5 principal components (PCA1.5)	0.2920	0.002	0.518
	PCA using principal components that explain 80% (PCA80)	0.00396	0.022	0.050

## Discussion

An extensive simulation study comparing a variety of self-contained gene set methods for analysis of gene expression data was carried out. Results demonstrated that among the methods considered, Fisher's method (FM) [12] and a global model with random effects (GMRE) [6] have the highest power to detect gene set effects. As compared to GMRE, the main advantage of FM is that it can be applied when only summary statistics or p-values for each gene expression probe are available. However, when original expression data are not available, the permutation-based p-value for the overall gene set effect cannot be obtained, and we must rely on p-values based on asymptotic theory. This can lead to an invalid test with inflated type 1 error when different expression values within a gene set are correlated. The main advantage of GMRE, on the other hand, is that it allows for estimation of effects in the context of other effects within the pathway, and inclusion of interaction effects. An investigation of the performance of various gene set approaches in detecting pathway effects in the presence of gene-expression interactions is warranted.

FM was more powerful than the other methods based on combining probe-specific p-values that were considered. FM treats p-values at the extreme of the distribution asymmetrically, being more sensitive to small p-values than large p-values. This leads to an advantage over methods such as Stouffer's Method (SM) [19] that avoids asymmetry by transforming p-values to normally distributed variables and then using them in a one-sided test. SM is therefore equally applicable to detecting deviations of p-values from the null distribution due to an overrepresentation of p-values close to 1, rather than p-values close to 0. However, as this is not the goal of the overall test of association of a pathway with the phenotype, this feature is not advantageous in this context. For large sample sizes (n≥100), the proposed MTS test statistic with the use of a log transformation of the p-value resulted in higher statistical power than the originally proposal TS measure. However, the MTS test still falls short of both FM and GMRE methods in terms of power.

Similar approaches can be considered in the context of analysis of genotype data from single nucleotide polymorphisms (SNPs) within genes that belong to a gene set. The key differences between expression and genotype gene set data is that SNP-sets tend to be much larger (more SNPs than expression probes map to a gene set), and for SNPs there is a greater extent of correlation within a gene set, resulting from linkage disequilibrium (LD) within genes. Also, SNP data are ordinal (0, 1, or 2 minor alleles for each SNP) and different ways of modeling such data can be considered (e.g., dominant, recessive genetic models).

In our simulation study we generated a continuous phenotype that depended on a portion of expression values according to a specific model. The methods investigated here, or simple modifications of these methods, could also be applied to other phenotypes, such as case-control status or time-to-event outcomes. The p-value combination methods are particularly easily modified, as the individual gene/probe set p-values can be based on any appropriate method, such as logistic regression for a case-control analysis.

The global model with fixed effects (GMFE) cannot be applied to data with a large number of gene expression probes (number of genes greater than number of subjects), due to the model being non-estimable. Otherwise, all methods considered are relatively easy to implement and computationally feasible. Although calculating permutation p-values, as opposed to p-values based on asymptotic distributions, is more time-consuming, we recommend the use of permutations when the gene expression levels within a gene set are correlated. Use of asymptotic distributions assumes independence of gene expression levels and can lead to highly inflated type 1 error rates when this is an invalid assumption. For instance, with p-values based on the asymptotic chi-squared distribution (detailed results not shown), the mean type I error rates for FM across the null simulation scenarios increased as the level of dependency between the genes increased, with mean error rates of 0.051, 0.11, and 0.182 for correlation of 0.0, 0.1 and 0.3, respectively. It should also be noted that if an appropriate testing method is not used to assess the association of each gene within the gene set with the phenotype, the aggregation of these results for determining the association of the gene set will be invalid. Thus, when applying FM for gene set analysis, one should choose an appropriate analysis method (and if possible, the most powerful) for assessing the association of each gene with the phenotype.

Application of the gene set methods to data from a pharmacogenomics study provided evidence that the expression of genes in the gemcitabine and glutathione pathways are associated with the IC_50_ outcome following treatment of cell lines with gemcitabine. The application of the various gene set methods to the gemcitabine pharmacogenomic study demonstrates one of the key advantages of self-contained methods: these methods can be applied to a specific candidate pathway, without requiring genome-wide data for other pathways. Analysis of specific pathways of interest can be more powerful as it reduces the need for correction for multiple testing.

The results obtained for the pharmacogenomics example are consistent with the prior knowledge that the gemcitabine pathway contains genes that play a role in individual response to the drug gemcitabine. However, not all the gene set methods were able to detect the association of the phenotype with this pathway. In particular, the KS, SM, TS, MTS, and PCA method using only the top component, did not detect a significant association at the 0.05 significance level. These results are consistent with our simulation study results, which demonstrated that these methods tended to have lower power than the other gene set tests. However, for the gemcitabine pathway, principal component analysis with the top 5 PCs detected stronger evidence of gene-set association than other approaches, even though in simulations this was generally not the most powerful approach. This is not surprising, as the actual power of each method for a given data set depends on the underlying situation, such as the number of expression probes truly associated with the outcome, the levels of association, etc. Perhaps for the gemcitabine pathway, the top five PCs were optimal for capturing the relevant variation in expression as related to the IC_50_ outcome. This is also observed in the analysis of the glutathione pathway, in which the GMFE approach produced the most significant results. This also agrees with the simulation study, in which, if possible to fit, the GMFE approach was one of the most powerful approaches. The simulation results provide an overall power comparison across methods for a wide range of situations.

In conclusion, over a variety of scenarios, the FM [12] with empirical p-values or the GMRE [6] were the most powerful analytical approaches for a self-contained gene set analysis. Therefore, we recommend either the FM [12] with empirical p-values or the GMRE [6] for the analysis of gene expression data with self-contained gene set analysis.

## Supporting Information

Table S1 Type I error rates for various simulations scenarios for all gene set methods considered (excel file).
(0.04 MB XLS)

Table S2 Power for various simulations scenarios for all gene set methods considered (excel file).
(0.47 MB XLS)

Figure S1 Plots of power for all methods as a function of the correlation between expression values within the gene set (ρ), the proportion of probes associated with the phenotype, and the calculated R2 (the proportion of variation in the quantitative phenotype explained by the gene expression values in the pathway). The average power values are based on all simulated non-null scenarios, with plots (A)–(C) being based on sample size of 20, and plots (D)–(F) being based on sample size of 500. The plots of power as a function of correlation (A and D) exclude scenarios with between-probe correlation structure defined by the gemcitabine pathway, and only shows fixed-correlation scenarios (ρ = 0, 0.1, 0.3). For plots (B), (C), (E) and (F) a kernel smoother was used to fit a curve to the data. Scenarios with all expression probes being associated with the trait were excluded from plot (B) and (E), as all the methods had very high power in this situation.
(0.31 MB EPS)
